# Case Report: Unilateral optic perineuritis as the initial presentation of multiple myeloma

**DOI:** 10.3389/fopht.2025.1616532

**Published:** 2025-11-06

**Authors:** Eugene Jung, Jae-Hwan Choi, Kwang-Dong Choi, Seo-Young Choi

**Affiliations:** 1 Department of Neurology, Pusan National University Hospital, Pusan National University School of Medicine and Biomedical Research Institute, Busan, Republic of Korea; 2 Department of Neurology, Pusan National University School of Medicine, Research Institute for Convergence of Biomedical Science and Technology, Pusan National University Yangsan Hospital, Yangsan, Republic of Korea

**Keywords:** multiple myeloma, optic neuritis, optic perineuritis, visual loss, optic nerve

## Abstract

**Background:**

Multiple myeloma (MM) is typically diagnosed based on systemic symptoms such as anemia or fatigue, or through imaging findings of bone lytic lesions. Neurologic manifestations as the initial presentation are rare. In this case, we report a patient diagnosed with MM after presenting with severe, unilateral vision loss.

**Methods:**

A 67-year-old woman presented with a 5-day history of painless vision loss in her left eye. Her visual acuity was severely reduced to light perception, yet both the optic disc and retinal vessels appeared normal upon examination. Blood tests revealed no significant abnormalities except for anemia. Magnetic resonance imaging (MRI) revealed enhancement of the left optic nerve sheath, and bone marrow biopsy confirmed MM. High-dose steroid and subsequent chemotherapy led to significant visual improvement.

**Conclusions:**

Unilateral optic perineuritis can be the first manifestation of MM, potentially caused by immune-mediated mechanisms or direct tumor cell infiltration near the optic nerve. Steroid not only contributed to symptom improvement but also may have played a role in partial remission of MM.

## Introduction

Multiple myeloma (MM) is a hematologic malignancy marked by abnormal plasma cell proliferation, commonly presenting with bone lesions, kidney injury, anemia, or hypercalcemia (1). While over 70% of cases are diagnosed through anemia or bone lytic lesions, only about 3% present as extramedullary disease, including nervous system involvement (1).

There are few reports with optic nerve involvement, and those were after the diagnosis of MM (2). The occurrence of acute ophthalmological symptoms as the first clue leading to a diagnosis of MM is exceedingly rare. Herein, we report an unusual case in which MM was diagnosed based on acute unilateral vision loss due to optic perineuritis.

## Case description and diagnostic assessment

**Figure 1:**
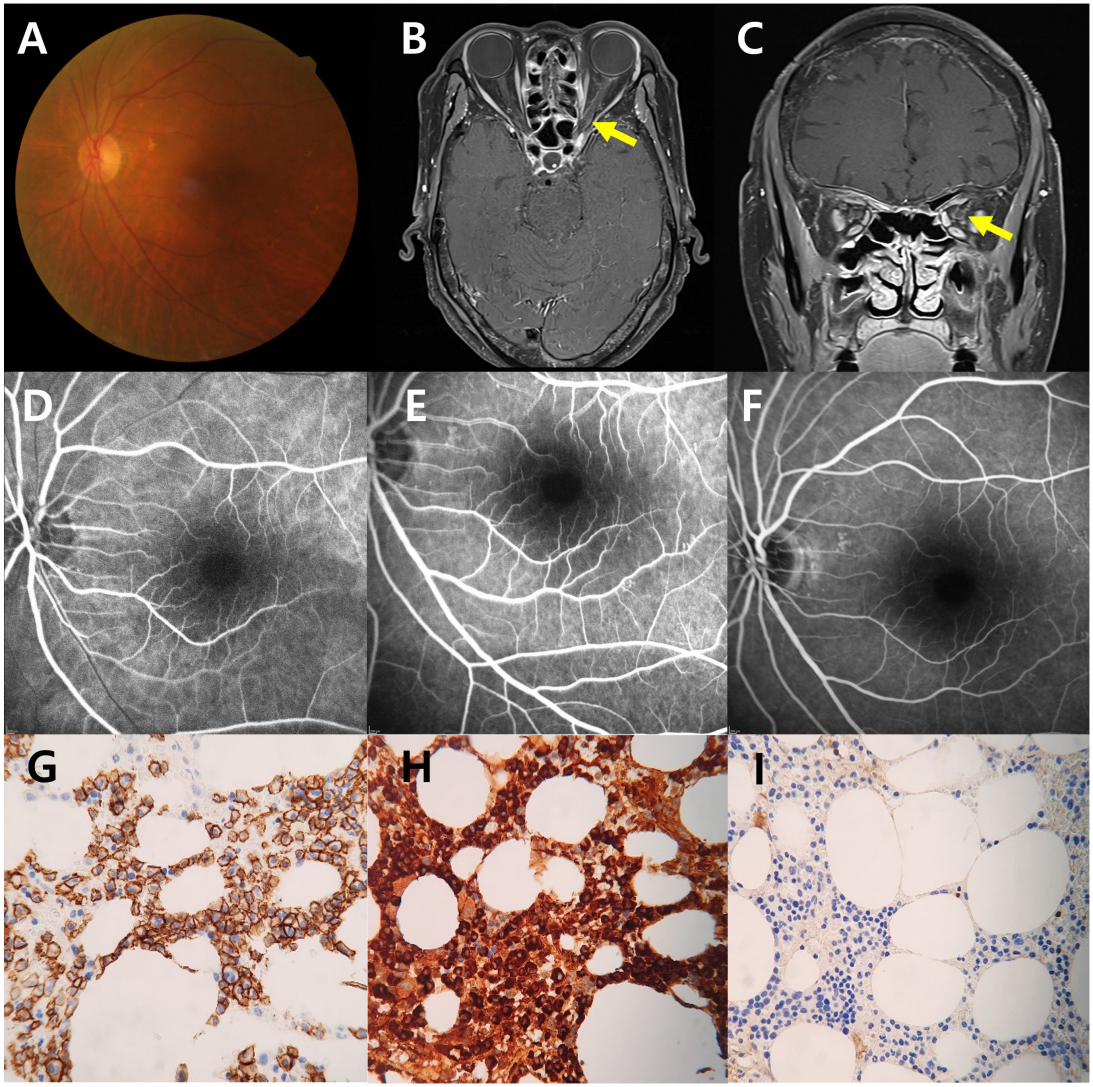
Fundus photography shows the normal optic disc of the left eye **(A)**. Axial **(B)** and coronal **(C)** fat-suppressed T1-weighted magnetic resonance images demonstrate the high signal intensity of the left optic nerve sheath with marked enhancement (arrows). Any compressive lesion, leptomeningeal enhancement, other brain lesions, or other bony lytic lesions were not revealed. Fluorescein angiography demonstrates normal findings in the early phase at 11 s **(D)**, arteriovenous phase at 20 s **(E)**, and late phase at 5 min 30 s **(F)**. Immunohistochemical analysis at 400× magnification of biopsy in colon demonstrates neoplastic plasma cells with strong, diffuse membranous positivity for CD138, appearing as an intense brown staining, a hallmark marker for plasma cells, indicating their neoplastic origin and widespread infiltration **(G)**. *In situ* hybridization targeting light chain mRNA reveals **(H)** kappa and **(I)** lambda expression. In **(H)**, kappa light chain restriction is highlighted by an intense dark brown signal, whereas **(I)** shows a minimal or absent brown signal, consistent with the monoclonal nature of the plasma cell population. Morphologically, the plasma cells exhibit eccentric nuclei, prominent perinuclear hof, and coarse chromatin, further emphasizing their neoplastic features.

**Figure 2:**
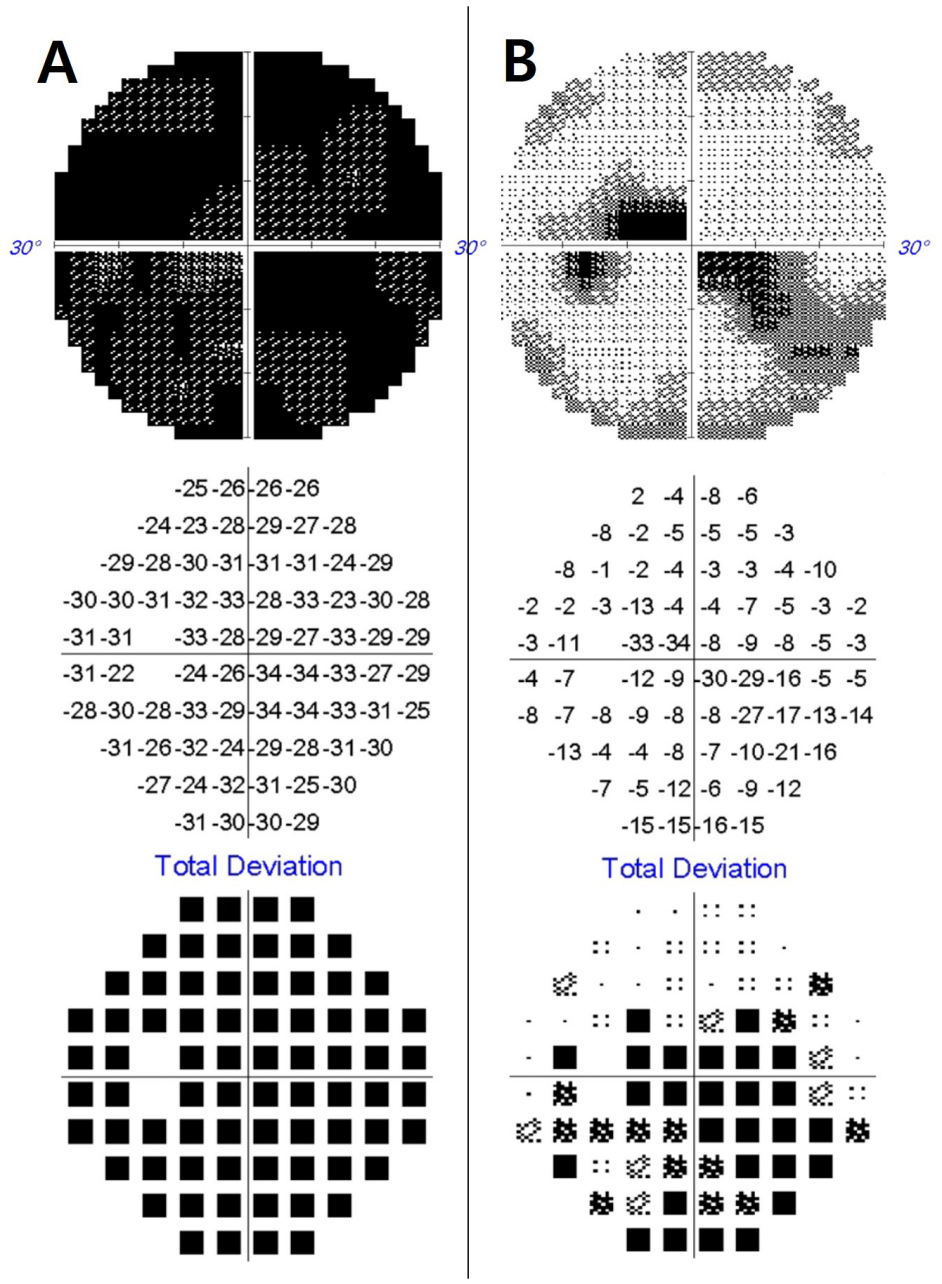
Initial Humphrey perimetry demonstrates a severe visual field defect in the left eye [mean deviation: –29.52 dB, **(A)**]. Following treatment, the visual field markedly improved, with a mean deviation of –10.13 dB **(B)**.

A 67-year-old woman presented with a 5-day history of painless vision loss in her left eye. Her medical history included angina pectoris and coronary artery bypass surgery. Corrected visual acuity prior to onset had been 20/40 in both eyes. On neurological examination, there were no accompanying sensory or motor deficits. Her visual acuity was limited to light perception in the left eye and 20/40 in the right. Fundus photography (**Figure 1A**), fluorescein angiography (**Figures 1D–F**), and optical coherence tomography were unremarkable. The Humphrey visual field test revealed a mean deviation (MD) of −29.52 dB (**Figure 2A**), indicating severe visual field loss. Orbital magnetic resonance imaging (MRI) revealed enhancement of the left optic nerve sheath without nerve involvement or compressive lesions (**Figures 1B,C**). Other meningeal or parenchymal abnormalities were not noted.

Laboratory evaluation showed marked anemia (hemoglobin, 6.8 g/dL). Fluorescent antinuclear antibodies; anti-Smith, anti-Ro, and anti-La; anti-nuclear and anti-dsDNA antibodies; and paraneoplastic antibodies including anti-Hu, anti-Yo, anti-Ri, anti-amphiphysin, anti-CV2, anti-Ma, anti-SOX1, and anti-recoverin were negative; angiotensin converting enzymes were within normal limit. The anti-aquaporin 4 IgG antibody and MOG antibody were also negative. Erythrocyte sedimentation rate was 88 mm/h (normal, 0–15) and C-reactive protein was 2.5 mg/dL (normal, 0–0.5). Cerebrospinal fluid analysis showed mild pleocytosis (14 cells/μL) and elevated protein (168.1 mg/dL) but no malignant cells or oligoclonal bands.

Chest computed tomography (CT) revealed post-status median sternotomy without any sarcoidosis. Abdominal CT revealed mural thickening of the transverse colon, with biopsy confirming light chain amyloidosis. Serum and urine protein electrophoresis showed IgG-kappa monoclonal protein. Bone marrow biopsy revealed >60% plasma cells demonstrated by CD138 immunohistochemical staining, confirming MM (**Figures 1D–F**). According to the Revised International Staging System and International Myeloma Working Group, the patient was also categorized as stage II, moderate stage, with a standard-risk profile.

## Discussion

In elderly patients with vascular risk factor, the most clinically significant consideration for painless severe visual loss is an ischemic lesion affecting the retina or optic nerve (3). Since arterial anterior ischemic optic neuritis (AION) is accompanied by pain, it can be excluded, but non-arterial AION can also commonly occur in patients such as ours. However, our patient did not exhibit any abnormalities in the retinal vessels or the optic disc, nor were there any findings suggestive of inflammation, making the diagnosis particularly challenging. Nevertheless, the thorough systemic evaluation from unusual optic perineuritis led to the timely diagnosis of a serious underlying malignancy. This case is unique in that unilateral optic perineuritis was the first and sole manifestation of MM. Although bilateral optic nerve involvement has been reported in MM, it usually occurs later in the disease course or as a manifestation of CNS spread (4). To the best of our knowledge, this is the first reported case in which painless unilateral optic nerve involvement led to the diagnosis of MM.

Potential mechanisms include direct neoplastic infiltration, immune-associated inflammation, or perineural amyloid deposition (3). Leptomeningeal seeding typically presents with headache, bilateral involvement, and dural thickening, allowing easier differentiation from other optic nerve disorders (5). However, painless unilateral optic perineuritis as observed in our case represents an atypical clinical presentation (6). When observing such atypical features, it may be considered that the inflammation is caused by immune-associated rather than idiopathic perineuritis, which is mainly accompanied by pain (6).

Steroids are a cornerstone treatment in both idiopathic perineuritis and MM regimens (1, 6). In our case, the marked improvement in visual function following steroid therapy may suggest immune-mediated pathology, but this also makes it difficult to exclude the possibility of direct involvement of MM (7). Even if it is important to rule out the possibility of a very rare disease occurring spontaneously, a biopsy is difficult to perform on the optic nerve because it may cause irreversible damage. Further analysis of similar cases in the future may be necessary to better understand this rare presentation.

## Patient perspective

The patient received intravenous methylprednisolone (1 g/day for 5 days), which resulted in rapid visual improvement. Her visual acuity recovered to 20/40 bilaterally, and visual field MD improved to −10.13 dB (**Figure 2B**). Following chemotherapy, the VRd regimen (bortezomib, lenalidomide, and dexamethasone) was initiated and continued for over 10 months, during which her vision remained stable.

## Data Availability

The raw data supporting the conclusions of this article will be made available by the authors, without undue reservation.
